# Liposarcome de la loge rénale: à propos de deux cas avec revue de la littérature

**DOI:** 10.11604/pamj.2018.29.167.1850

**Published:** 2018-03-22

**Authors:** Rajae Tahri, Lamiaa Gamra, Azzedine El Otmany

**Affiliations:** 1 Centre Hospitalo-universitaire Ibn Sina, Faculté de Médecine et de Pharmacie, Rabat, Maroc

**Keywords:** Liposarcoma in the renal compartment, tumor, ultidisciplinary management, specialized, Liposarcoma in the renal compartment, tumor, ultidisciplinary management, specialized

## Abstract

Les sarcomes rétropéritonéaux sont des tumeurs rares et hétérogènes. Nous en présentons deux cas localisés à la loge rénale avec revue de la littérature dans le but d’insister sur les particularités anatomiques et chirurgicales de cette localisation, et sur ses éventuels retentissements pronostiques. Le premier cas de tumeur a été rapporté chez une patiente de 45 ans localisée au niveau de la loge rénale gauche, dont le diagnostic de liposarcome de type myxoïde a été posé sur l’examen anatomo-pathologique de la pièce de résection. Le deuxième cas de tumeur a été rapporté, chez un homme de 70 ans, localisé au niveau de la loge rénale droite, dont le diagnostic de liposarcome dédifférencié a été posé sur l’examen anatomo-pathologique de la pièce de résection. Les sarcomes rétropéritonéaux sont souvent diagnostiqués au stade de masse palpable. Le traitement de référence est la résection tumorale complète avec des marges saines et sans effraction. Dans ce but un élargissement aux organes de proximité est recommandé par certains auteurs. Cependant les contraintes anatomiques de l’espace rétropéritonéal et le volume tumoral souvent important limitent les possibilités d’avoir une clairance satisfaisante. Le scanner et l’imagerie par résonance magnétique sont d’un grand apport. Le diagnostic histologique pose des problèmes, ainsi le recours aux techniques d’immunohistochimie et parfois de biologie moléculaire est d’une grande aide. L’évolution est marquée par la survenue fréquente de récidives. Afin d’optimiser les résultats thérapeutiques de ces tumeurs rares et très variés, une prise en charge multidisciplinaire spécialisée est recommandée.

## Abstract (en)

Retroperitoneal sarcomas are rare and heterogeneous tumors. We here report two cases of liposarcoma in the renal compartment with a literature review in order to emphasize on the anatomical and surgical features of this tumor location and on its possible prognostic implications. The first reported case was a 45-year old female patient with liposarcoma at the level of the left renal compartment. The diagnosis of myxoid liposarcoma was based on anatomo-pathological examination of the surgical specimen. The second reported case was a 70-year old man with liposarcoma at the level of the right renal compartment. The diagnosis of dedifferentiated liposarcoma was based on anatomo-pathological examination of the surgical specimen. Retroperitoneal sarcomas are often diagnosed with a palpable mass. The gold standard treatment is complete tumor resection with healthy resection margins and without damage or rupture. For this purpose resection involving adjacent organs is recommended by some authors. However the anatomical constraints of tretroperitoneal compartment and the often important tumor volume limit the possibilities to achieve a satisfactory resection. CT scan and MRI are very useful imaging techniques. Histological diagnosis poses some problems; therefore immunohistochemistry and sometimes molecular biology are significantly helpful. Patient evolution is marked by the frequent occurrence of recurrences. A specialized multidisciplinary management of these rare and very varied tumors is recommended in order to optimize therapeutic outcomes.

## Introduction

Les sarcomes sont des tumeurs malignes rares. Ils siègent au niveau des tissus mous dans plus de 50% des cas [1]. Les sarcomes des tissus mous (STM) représentent 1% des tumeurs des tissus mous, moins de 1% de tous les cancers de l'adulte, et à peu près 15% de ceux de l'enfant [2, 3]. Les sarcomes rétropéritonéaux (SRP) sont des tumeurs rares et hétérogènes. Ils représentent 15% des sarcomes de tissus mous, avec une incidence annuelle de 1000 aux État-Unis et de 300 en France [4,5]. Le traitement fondamental est une chirurgie complète avec marges saines et sans effraction, mais celle-ci est souvent difficile. La qualité de l'exérèse est le facteur pronostique déterminant retrouvé dans la littérature [6-8]. Le pronostic de ces tumeurs reste défavorable en rapport avec un taux élevé des récidives locorégionales. Le taux de survie globale à cinq ans de tous les SRP ne dépasse pas 15 à 30% [9]. Afin d'optimiser les résultats thérapeutiques de ces tumeurs rares, une prise en charge multidisciplinaire est obligatoire. Nous allons présenter notre expérience de deux cas de sarcomes géants de la loge rénale colligés à l'Institut National d'Oncologie de Rabat afin de souligner les particularités anatomiques et chirurgicales de cette localisation au sein du rétropéritoine, et ses éventuels retentissements pronostiques, et afin de retenir les sarcomes de cette localisation comme entité à part au sein des sarcomes rétropéritonéaux.

## Patient et observation

### Observation n°1

**Figure 1:**
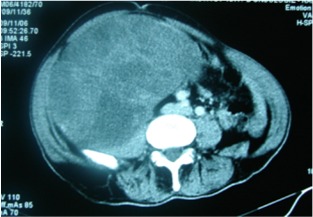
Coupe TDM montrant un processus tumoral bien limité, refoulant et écrasant le rein gauche en dedans et en avant, se rehaussant de façon hétérogène, développé au dépend de la loge rénale gauche

**Figure 2:**
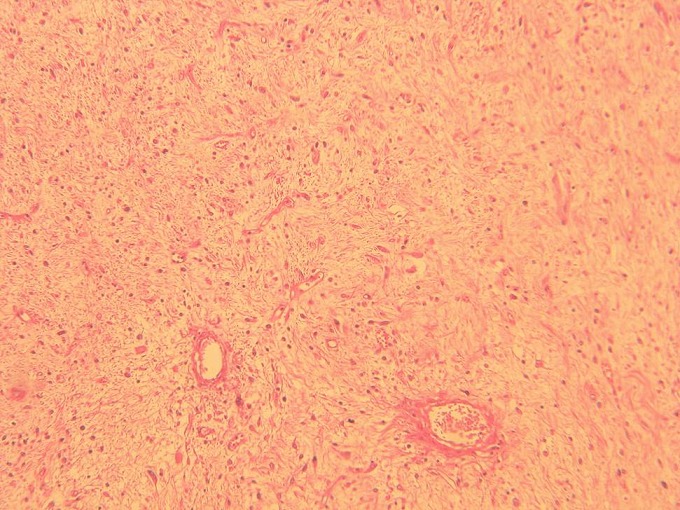
Prolifération tumorale faite de cellules allongées se développant au sein d’un stroma fibreux avec une trame vasculaire cernée de coulées inflammatoire (HEx40)

**Figure 3:**
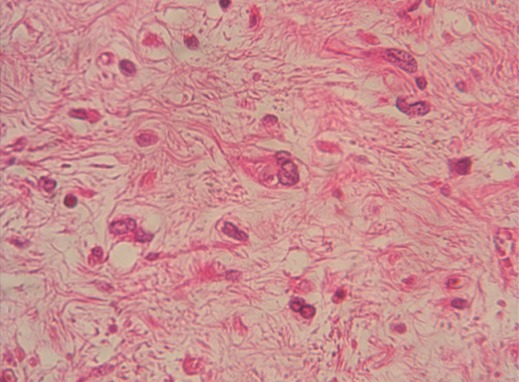
Présence de cellules monstrueuses munies de noyaux volumineux très irréguliers à chromatine granuleuse parfois granuleuse (HEx400)

**Figure 4:**
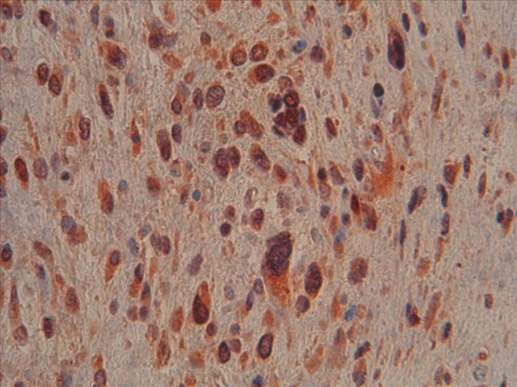
Marquage MDM2 positif des cellules monstrueuses (Gx400)

Il s'agit d'une patiente âgée de 45ans, sans antécédents pathologiques notables. Le début de sa symptomatologie remontait à 10mois avant la consultation par l'apparition de lombalgies et d'une masse au niveau du flanc gauche augmentant progressivement de volume, associés à une pollakiurie, sans autres signes urinaires, ni digestifs, ni neurologiques, le tout évoluant dans un contexte d'amaigrissement non chiffré. L'examen clinique avait trouvé une patiente en bon état général, abdomen distendu. A la palpation, la masse prenait toute la partie gauche abdomino-pelvienne et dépassait la ligne médiane vers la droite, de surface lisse et de consistance ferme. Aux touchers pelviens, le pôle inférieur de la masse comblait le pelvis. Le reste de l'examen somatique n'avait pas noté de particularités. La Tomodensitométrie abdomino-pelvienne réalisée avait retrouvée une énorme masse bien limitée occupant l'hémi-abdomen gauche, mesurant 14x19x20cm, et étendue de la 10^ème^ côte jusqu'au détroit supérieur (Figure 1). Elle écrase et refoule le rein en dedans qui dépasse la ligne médiane d'environ 5cm. Elle exerce un effet de masse sur les structures de voisinage sans signes d'envahissement. Elle est de densité tissulaire hypodense se rehaussant de façon hétérogène après injection de produit de contraste et sans signe de nécrose. Cet aspect évoque la nature adipeuse maligne du processus développé au niveau de la loge rénale. La radiographie du thorax était normale. Sur ces données, les points discutés sur le plan technique sont: comment avoir une bonne exposition opératoire et comment aborder l'artère rénale du fait du volume tumoral important et du mode de développement de la masse en contact du pédicule rénal. La patiente a été opérée par une laparotomie médiane xyphopubienne, élargie par un refend transversal gauche. A l'exploration, la tumeur occupait l'étage sus et sous-mésocolique gauche, et plongeait dans le pelvis en dépassant la ligne médiane vers la droite. Le temps clef de l'intervention était, après décollement du côlon gauche, la libération du pôle inférieur de la tumeur permettant le repérage et le contrôle de l'artère rénale avant toute dissection de la veine rénale gauche moulée sur la tumeur. La découverte des adénomégalies lombo-aortiques gauches a justifié, en l'absence d'un diagnostic histologique, un curage lombo-aortique associé à l'exérèse de la loge rénale du côté gauche. L'intervention avait duré 3h20min et le saignement a été évalué à 200ml. L'examen anatomopathologique avait porté sur une pièce d'exérèse tumorale élargie prenant la totalité de la loge rénale et son contenu, pesant 4900g et mesurant 29x21x17cm. La pièce est bien encapsulée, de couleur rose avec une vascularisation bien développée en surface. A la coupe on notait la présence d'une énorme tumeur de couleur rose-grisâtre qui refoule et écrase le rein en périphérie. Celui-ci mesure 14x5,5x6cm, avec une surrénale réduite en un liseré de 6x2x0,3cm. L'uretère repéré mesure 10cm de long. Le curage, renfermait 20 ganglions dont le plus volumineux mesure 1,5cm de grand axe. L'étude morphologique des différents prélèvements réalisés au niveau de la tumeur, montre une prolifération tumorale de densité cellulaire variable. On y observe des territoires riches en cellules aux noyaux allongés ou arrondis à chromatine dense; en d'autres points les cellules sont dissociées par l''dème dans une toile conjonctive disloquée ou parfois dense. Les noyaux sont hyperchromatiques avec dyscaryose modérée. On note en plusieurs endroits des cellules monstrueuses munies de noyaux volumineux très irréguliers, ou plurinucléés à chromatine granuleuse, avec gros nucléoles proéminents MDM2 positives (Figure 2, Figure 3, Figure 4). Le stroma est tantôt fibreux dense, tantôt myxoïde avec présence de nombreux adipocytes matures prédominants en périphérie. Il s'y associe une abondante trame vasculaire cernée de coulées inflammatoires polymorphes lympho-plasmo-histiocytaires. Le processus sus décrit infiltre la capsule et le parenchyme rénaux. La voie excrétrice, les vaisseaux et la surrénale ne sont pas envahis. La capsule externe de la loge rénale est intacte. Les 20 ganglions examinés sont indemnes de toute lésion tumorale. En conclusion, l'aspect morphologique et immunohistochimique d'un liposarcome de type myxoïde. L'exérèse est complète, la capsule externe de la loge rénale et les ganglions sont intacts. Aucune thérapeutique adjuvante n'a été jugée nécessaire. La patiente est suivie régulièrement selon un rythme d'un examen clinique chaque 4mois et d'une TDM abdomino-pelvienne chaque 6mois. Le recul est de 43mois sans signes de maladies décelables.

### Observation n°2

**Figure 5:**
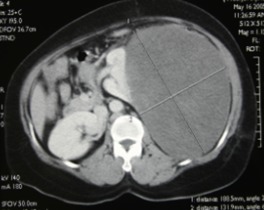
Coupe TDM montrant une tumeur hypodense bien limitée refoulant le rein droit en dehors et en avant contre la paroi abdominale antérieure

Il s'agit de Monsieur A.A, âgé de 70 ans, fellah de profession, sans antécédents pathologiques particuliers, suivi à l'Institut National d'Oncologie à Rabat depuis le 05 Octobre 2006. Le début de sa symptomatologie remontait à 06mois avant sa première consultation par l'apparition d'une masse abdomino-pelvienne douloureuse au niveau du flanc droit augmentant progressivement de volume sans signes urinaires, ni digestifs, ni neurologiques associés, le tout évoluant dans un contexte d'amaigrissement non chiffré. L'examen clinique avait noté une déformation abdominale prédominant du côté droit, la palpation abdominale et le toucher rectal avaient retrouvé une masse abdomino-pelvienne ferme de surface lisse étendue de l'hypochondre droit jusqu'au pelvis et dépassant la ligne médiane de 8cm vers la gauche, fixe par rapport au plan postérieur. Les aires ganglionnaires étaient libres. L'examen des testicules et le reste de l'examen somatique étaient sans particularités. L'hémoglobine était à 9,6g/dl. L'échographie évoquait une tumeur rénale. La radiographie thoracique était normale. A la TDM, la tumeur était bien limitée, prenant la totalité de l'hémi-abdomen droit et arrivant jusqu'au détroit supérieur. Elle dépasse la ligne médiane, et mesure 22x16cm. Elle refoule le rein en dedans et en avant en le dysrotant et le plaquant contre la paroi abdominale antérieure (Figure 5). Elle exerce un effet de masse sur les structures avoisinantes en particulier sur la veine cave inférieure VCI non visualisée dans sa quasitotalité. Elle est de densité tissulaire comportant des plages hypodenses et se rehaussant de façon hétérogène à l'injection du produit de contraste. Cet aspect évoque un processus de la loge rénale dont la nature liposarcomateuse est très probable. Sur ces données les points discutés sur le plan technique sont: l'exposition du champ opératoire du fait du volume et du mode de développement tumoral, l'identification et la libération de la VCI, surtout qu'elle est refoulée et aplatie dans sa totalité et que les veines iliaques -qui peuvent servir comme repère- sont cachées par la tumeur, et enfin le contrôle du pédicule rénal. Opéré le 12 Décembre 2006, par une laparotomie médiane xyphopubienne élargie par double refends transversaux bilatéraux. A l'exploration, la tumeur écrase le foie en haut, plonge dans le pelvis en bas et dépasse la ligne médiane de 6cm. La VCI était non identifiable et la dissection de la veine rénale droite était la seule possibilité de la repérer. Elle était collabée et moulée sur la masse. La dissection de sa partie sous rénale et l'extériorisation du pôle inférieur de la masse ont permis de contrôler le pédicule rénal et de compléter la libération de la VCI vers le haut. L'intervention est terminée par une résection de la loge rénale élargie au fascia iliaca. L'opération a duré 4heures et demi, le saignement en peropératoire est évalué à moins de 250ml et les suites opératoires étaient simples.

**Figure 6:**
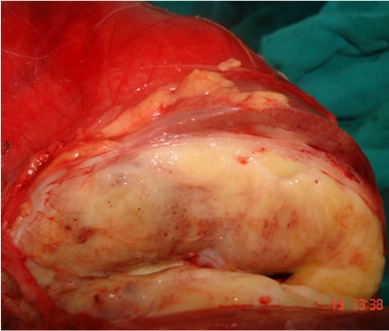
Pièce opératoire ouverte montrant le fascia périrénal, le rein écrasé, la pseudocapsule tumorale et la tumeur

L'examen anatomopathologique de la pièce opératoire trouve: 1- à l'examen macroscopique, une pièce d'exérèse tumorale élargie prenant la totalité de la loge rénale et son contenu. Elle pèse 6kg et mesure 29x17x16,5cm. L'aspect externe de la pièce est bien encapsulé. A la coupe, on note la présence d'une énorme tumeur de couleur blanc-grisâtre qui refoule et écrase le rein en périphérie. La surrénale réduite en un liseré de 5x1,2x0,3cm. L'uretère repéré mesure 14cm de long. Le prélèvement ganglionnaire inter-aortico-cave et iliaque primitif droit renfermait 7 ganglions dont le plus volumineux mesure 2cm de grand axe (Figure 6); 2- histologiquement, les différents prélèvements réalisés au niveau de la tumeur répondent à une double prolifération tumorale. Celle qui prédomine est faite de cellules fusiformes au cytoplasme éosinophile peu abondant et aux noyaux allongés hyperchromatiques anisocaryotiques avec des atypies légères ou modérées. L'autre prolifération est faite de secteurs de liposarcome bien différencié constitué de cellules adipocytaires aux noyaux hyperchromatiques atypiques et de rares lipoblastes. Cette tumeur comprime le parenchyme rénal sans l'envahir et respecte la capsule, les voies excrétrices, le hile rénal et la surrénale. La recoupe urétérale, le fascia iliaca, la capsule externe de la loge rénale et les 7 ganglions examinés sont indemnes de toute lésion tumorale. En conclusion, l'aspect morphologique est celui d'un liposarcome dédifférencié. Aucun traitement adjuvant n'a été associé. Le patient est suivi régulièrement en consultation sans signe de maladie clinique ni radiologique avec un recul de 26 mois.

## Discussion

La majorité des tumeurs rétropéritonéales sont malignes et approximativement 30% sont des sarcomes [10]. Le liposarcome représente environ 40 à 50% des sarcomes rétropéritonéaux. Le liposarcome rétropéritonéal (LRP) atteint de façon égale les deux sexes. L'âge moyen au moment du diagnostic se situe vers la cinquième décade, mais la maladie peut intéresser toutes les tranches d'âges [11,12]. Nos deux cas de LRP sont survenus à 45ans chez la femme et à 70ans chez l'homme. L'augmentation lente de la taille tumorale et la compliance de l'espace rétropéritonéal expliquent le caractère pauci-symptomatique et le volume important de la tumeur au moment du diagnostic (pouvant atteindre 40kg) [10-14]. Les symptômes révélant ces tumeurs ne sont pas spécifiques. Ils sont dus généralement à la compression des organes avoisinants. Ils sont dominés par la douleur ou la pesanteur abdominale (50 à 80%) et par la perception d'une masse abdominale (70 à 80%). Plus rarement, ces signes s'associent à des troubles urinaires et/ou digestifs, à une altération de l'état général et à une fièvre secondaire à la nécrose tumorale [11, 15]. Le liposarcome rétropéritonéal révélé par une complication inaugurale telle qu'une hémorragie, une occlusion intestinale, une perforation ou un volvulus a exceptionnellement été rapporté dans la littérature [13]. Les localisations multiples synchrones sont exceptionnelles et rapportées sous forme de faits cliniques dans la littérature. La distinction entre des localisations secondaires dont la lésion primitive reste à rechercher et une origine multicentrique de la tumeur est difficile [16-18]. Dans nos deux cas, le motif de consultation était des douleurs abdominales avec une énorme masse abdominale de 4,9kg et 6kg. La TDM et l'imagerie par résonance magnétique (IRM) abdomino-pelviennes permettent de lier la masse au rétropéritoine, de faire une approche diagnostique de nature, de réaliser le bilan d'extension locorégional, le bilan d'extension à distance, de mieux cibler les prélèvements percutanés, et de planifier la stratégie opératoire. La nature lipomateuse de la lésion peut être suggérée avec une grande spécificité. La distinction entre lipome et liposarcome bien différencié constitue un dilemme fréquent en raison des similitudes radiologiques entre ces deux lésions. Certaines caractéristiques radiologiques permettent, cependant, de les différencier. En dehors de la taille importante de la lésion, la présence de septas épais, de lésions nodulaires et/ou globuleuses, et de zones non graisseuses ainsi que la diminution de la composition adipeuse de la lésion orientent fortement vers le liposarcome [16]. A l'échographie seule, il est difficile de préciser l'origine de la lésion; c'était le cas de la patiente HR qui nous a été adressée suite à une laparotomie exploratrice sur diagnostic échographique de tumeur de l'ovaire et du patient A.A. qui nous a été confié avec le diagnostic échographique de tumeur rénale.

Dans nos deux cas, le complément scannographique abdomino-pelvien réalisé, nous a permis de retenir le diagnostic de tumeur rétropéritonéale, et plus précisément de tumeur de la loge rénale et d'évoquer sa nature adipeuse agressive, de retenir la faisabilité technique de l'intervention et enfin d'établir une stratégie opératoire pour chacun de nos deux patients. Concernant la biopsie percutanée préopératoire des tumeurs rétropéritonéales; tous les auteurs s'accordent pour recommander une biopsie diagnostique lors de: tumeur non résécable, de suspicion de tumeur métastatique ou de lymphome et quand un traitement néoadjuvant est envisagé avant l'exérèse. Cependant, elle est discutée dans le cas d'une tumeur dont l'exérèse complète semble envisageable. Certains auteurs la recommandent systématiquement, par voie rétropéritonéale à l'aiguille protégée pour limiter au maximum le risque de contamination [19,20]. Dans ces cas la biopsie permettrait de redresser le diagnostic dans 5 à 10% des cas [19]. Aucun de nos deux patients n'a bénéficié de biopsie préopératoire qu'on a jugé inutile du fait qu'elle ne va pas changer notre conduite thérapeutique et à risque du fait qu'elle va communiquer la tumeur avec le RP. L'examen anatomopathologique de la pièce de résection tumorale permet, de juger la qualité de l'exérèse chirurgicale et de typer, sous typer et grader le sarcome. Concernant le liposarcome rétropéritonéal, plusieurs variétés histologiques de malignité croissante ont été décrites: Le liposarcome bien différencié, le liposarcome myxoïde, le liposarcome pléomorphe, le liposarcome à cellules rondes et le liposarcome dédifférencié [21]. Le liposarcome bien différencié, de meilleur pronostic représente 30 à 35% de l'ensemble des liposarcomes. Il peut récidiver localement après exérèse, mais il a un pouvoir métastatique faible. La forme myxoïde qui constitue la forme anatomopathologique la plus fréquente (50%) est cliniquement plus agressive, récidive rapidement et est de pronostic plus mauvais. Le liposarcome pléomorphe, le liposarcome à cellules rondes (qui représentent à eux deux, 10 à 15%des LRP) ainsi que le liposarcome dédifférencié sont de pronostic sombre [13]. Une association de deux ou trois types histologiques au sein de la même tumeur est rare (5 à 10%) réalisant le liposarcome de type mixte [14, 16]. Les localisations multiples du liposarcome rapportées dans la littérature sont constituées du même type histologique. Elles sont plus fréquentes lorsqu'il s'agit du type myxoïde [10, 15]. Par ailleurs, le liposarcome peut être associé à des tumeurs sarcomateuses d'un autre type ou à des lésions bénignes lipomateuses [16].

La dédifférenciation du liposarcome bien différencié est une complication reconnue, qui augmente avec la durée d'évolution de la tumeur. Des modifications génotypiques et caryotypiques ont été incriminées dans la dédifférenciation tumorale. Il est parfois difficile de différencier entre certains types de lipomes et les liposarcomes essentiellement bien différenciés, en se basant uniquement sur l'aspect morphologique. L'analyse chromosomique ainsi que les études cytogénétiques et moléculaires sont actuellement d'un grand apport pour le diagnostic différentiel des tumeurs adipeuses. La majorité des lipomes typiques sont caractérisés par la translocation t(3;12) (q27:q13-14) affectant le gène HMGIC [17]. La perte de la séquence 16q ou 13q est observée dans les lipomes pléomorphes et les lipomes à cellules fusiformes [18]. Les liposarcomes bien différenciés sont, quant à eux reconnus par la présence de chromosomes surnuméraires ayant pour principales caractéristiques, une forme circulaire en anneau et une taille anormalement importante. Il est actuellement bien établi que ces chromosomes surnuméraires sont constitués d'une amplification de la séquence 12q14-15 du bras long du chromosome 12. L'amplification du gène MDM2 est quasi constante alors que celle des gènes SAS, CDK4 et HMGIC est plus rare [19]. Les liposarcomes myxoïdes et à cellules rondes seraient liés à la translocation inverse t(12;16) (q13;p11) [22,23]. Chez nos patients, l'examen anatomopatholgique a retenu le diagnostic de liposarcome de la loge rénale, dédifférencié du côté droit et bien différencié du côté gauche, avec des marges non envahies dans les deux cas. L'arme thérapeutique principale des sarcomes rétropéritonéaux y compris les récidives est l'exérèse tumorale complète avec des marges saines et sans effraction [14,24,25]. La réussite de cette chirurgie est dépendante de l'expérience du chirurgien dans cette pathologie, de la qualité technique et analytique de l'imagerie médicale, et de la bonne planification technique de l'intervention.

De point de vue technique, la voie d'abord doit être bien réfléchie afin d'assurer une bonne exposition des temps opératoires à risque élevé sans trop d'attraction et de permettre d'enlever la tumeur en monobloc. Ceci exige des voies d'abord larges et parfois combinées particulièrement en cas d'extension dans un défilé anatomique. Dans nos deux cas la voie d'abord utilisée était une médiane xypho-pubienne élargie par un refend transversal gauche dans le cas de la tumeur de la loge rénale gauche, et par deux refends transversaux gauche et droit dans le cas de la tumeur du côté droit. La résection complète de ces tumeurs peut être une résection complète simple équivalente à l'énucléation imposée par le terrain ou les rapports anatomiques de la tumeur, une résection élargie de principe qui diminue le risque d'effraction et de marges envahies et qui est recommandée par certains auteurs, et enfin la résection élargie de nécessité où l'exérèse associée d'organes est imposée par l'infiltration tumorale. Du fait des contraintes anatomiques de l'espace rétropéritonéal (espace large et communiquant, refermant des organes vitaux et sans barrières anatomiques) et de la taille tumorale souvent importante, les marges chirurgicales sont toujours faibles ou nulles. La loge rénale, bien décrite par les anatomistes, est totalement limitée par le fascia périrénal (barrière anatomique) qu'on peut visualiser à l'imagerie médicale. Son importance est reconnue dans la pathologie infectieuse, traumatique, et néoplasique du rein. Elle nous paraît la seule localisation de sarcome au niveau du rétropéritoine où une exérèse tumorale peut respecter les principes de la chirurgie de ces tumeurs. Aussi le fait d'avoir la tumeur enfermée dans une loge fibreuse (la loge rénale) limite l'extension, diminue le risque d'effraction, facilite la dissection au contacte de la masse, et assure une qualité macroscopique et microscopique des marges. Sur la base du diagnostic de tumeur de la loge rénale chez nos deux patients, on n'a pas pratiqué de biopsie. Le geste chirurgical était une exérèse de la loge rénale (masse tumorale) sans élargissement vers les organes adjacent. Dans le cas de la tumeur de la loge rénale droite, toute la difficulté a résidé dans l'identification et la libération de la veine cave inférieur moulée sur la tumeur. Dans le cas de la tumeur de la loge rénale gauche, la résection a nécessité le décollement du côlon descendant, de la queue et du corps du pancréas et aussi de la rate au contact de la masse tumorale. Dans les deux cas les marges de résection étaient microscopiquement saines grâce au fascia périrénal qui constitue une barrière anatomique. Aucun de nos patients n'a bénéficié d'une thérapeutique adjuvante pour deux raisons: L'intérêt des traitements adjuvants n'est pas clairement retenu dans les SRP [12,14] et l'exérèse de la loge rénale est qualifiée équivalente à la compartimentectomie au niveau des membres (du fait de la présence du fascia périrénal fermant complètement la loge rénale).

Les tumeurs de la loge rénale peuvent être suspectées au scanner et/ou à l'IRM par le caractère bien limité, le refoulement, l'écrasement et la dystorsion précoce du rein. Mais aussi par la reconnaissance du fascia périrénal actuellement possible avec l'amélioration de la résolution spatiale des nouveaux scanners. Et elles sont confirmées à l'étude anatomopathologique de la pièce opératoire par la présence du fascia périrénal qui entoure l'ensemble tumeur-rein et qui constitue une barrière anatomique (Figure 6). L'intérêt de reconnaître cette entité de sarcome rétropéritonéal en préopératoire est d'éviter les biopsies qui vont communiquer la tumeur avec l'espace RP, de limiter les résections élargies de principe qui n'ont pas d'intérêt dans cette localisation et de pouvoir disséquer les organes au contact de la masse tumorale avec plus d'aisance et moins de risques carcinologiques (effraction tumorale ou marges envahies). Le problème majeur des sarcomes rétropéritonéaux réside dans l'échec du contrôle local (le taux de récidive locale est de 65% à 5ans). Le taux de résection complète varie selon les séries de 38 à 74% [6, 26]. La qualité de l'exérèse est constamment le facteur pronostique déterminant dans toutes les études multifactorielle [27, 28]. Actuellement, tous les auteurs s'accordent sur l'importance d'avoir des tranches de résection saines et de proscrire tout geste d'énucléation de la tumeur [27, 28]. Singer et al. ont montré que le taux de survie à cinq ans était de 70% après résection microscopiquement complète (R0), 41% après résection microscopiquement incomplète (R1) et 16% seulement en cas de résection macroscopiquement incomplète (R2) [28]. Chez nos deux patients, on souligne la qualité et la négativité des marges de résection (le fascia périrénal constitue une barrière anatomique résistante à l'infiltration tumorale). Le grade constitue le deuxième facteur pronostique indépendant retrouvé dans la littérature. Il permet d'évaluer le risque de récidive locale et de dissémination métastatique [12, 27,29]. Singer et al. ont montré que le risque de décès était multiplié par huit chez les malades qui avaient un sarcome rétropéritonéal de haut grade par rapport à ceux qui avaient un sarcome de bas grade [28]. Dans une étude rétrospective portant sur 40 patients traités d'un sarcome rétropéritonéal dans quatre centres de Languedoc, le haut grade (grades 2 et 3), l'atteinte des tranches de section et la bilatéralité étaient des facteurs pronostiques indépendants de récidive locale [30]. Le risque majeur des sarcomes rétropéritonéaux est la récidive locale et les patients décèdent le plus souvent de cette évolution locale. Le taux des récidives locales ou péritonéales varie de 44 à 85% à 5ans [29, 30]. Le traitement des récidives reste une chirurgie itérative après une exploration radiologique, quand une exérèse macroscopiquement complète est possible [31]. Ces récidives, au fil du temps, sont plus infiltrantes, plus agressives et souvent associées à une sarcomatose. Le contrôle locale est assuré chez nos deux patients avec un recul de 26 mois et de 43 mois. Le taux de survie globale à cinq ans est d'environ 50% [6, 29]. A l'exception de quelques équipes ayant essayé d'étudier l'impact de la localisation des sarcomes au niveau de RP sur le pronostic; aucune n'a étudié les particularités anatomiques, chirurgicales et pronostiques de la localisation des sarcomes au niveau de la loge rénale. L'apport de la radiothérapie n'est pas encore clair. Cette radiothérapie postopératoire diminue le risque de récidive locale et de décès [27, 32, 33]. L'étude réalisée par la FNCLCC a porté sur 145 patients traités d'un sarcome rétropéritonéal, a montré une réduction du risque de récidive locale par un facteur 3 chez les patients qui ont bénéficié d'une radiothérapie après une chirurgie complète [12]. Ce bénéfice de la radiothérapie postopératoire n'a pas été démontré dans d'autres études [10, 34]. L'inconvénient majeur reste la toxicité digestive. Cette toxicité est prévenue par le refoulement du tube digestif soit par une épiplooplastie, soit par la mise en place d'une prothèse [35]. Actuellement, la radiothérapie est préférée en situation préopératoire [36, 37]. Elle doit être réalisée avec des photons de haute énergie (6 MV) selon un fractionnement classique et avec une dose totale de 45 à 50Gy [36]. Il n'a pas été démontré qu'une chimiothérapie adjuvante allonge la survie. En situation néoadjuvante, la chimiothérapie est indiquée dans les sarcomes localement évolués et de haut grade. Elle peut permettre une réduction du volume tumoral et faciliter une chirurgie plus conservatrice et complète [38].

## Conclusion

Les sarcomes rétropéritonéaux sont des tumeurs rares et de pronostic défavorable. Une chirurgie complète est formellement recommandée afin de diminuer le risque de récidive locale et de métastase. La place de la radiothérapie et de la chimiothérapie néoadjuvante est en cours d'évaluation. Une prise en charge multidisciplinaire par une équipe spécialisée est recommandée. Face à l'absence de frontières ou de barrières anatomiques en dehors de la loge rénale dans le rétropéritoine et à la taille importante de ces sarcomes, la réussite de résection avec marges microscopiques non envahies est difficile à obtenir même en présence de résections macroscopiquement complètes.

## Conflits d’intérêts

Les auteurs ne déclarent aucun conflit d'intérêts.
